# Midbody-Localized Aquaporin Mediates Intercellular Lumen Expansion During Early Cleavage of an Invasive Freshwater Bivalve

**DOI:** 10.3389/fcell.2022.894434

**Published:** 2022-06-14

**Authors:** Elisabeth Zieger, Thomas Schwaha, Katharina Burger, Ina Bergheim, Andreas Wanninger, Andrew D. Calcino

**Affiliations:** ^1^ Integrative Zoology, Department of Evolutionary Biology, University of Vienna, Vienna, Austria; ^2^ Molecular Nutritional Science, Department of Nutritional Sciences, University of Vienna, Vienna, Austria

**Keywords:** lumenogenesis, midbody, blastomere cleavage, aquaporin, osmoregulation, freshwater invertebrate

## Abstract

Intercellular lumen formation is a crucial aspect of animal development and physiology that involves a complex interplay between the molecular and physical properties of the constituent cells. Embryos of the invasive freshwater mussel *Dreissena rostriformis* are ideal models for studying this process due to the large intercellular cavities that readily form during blastomere cleavage. Using this system, we show that recruitment of the transmembrane water channel protein aquaporin exclusively to the midbody of intercellular cytokinetic bridges is critical for lumenogenesis. The positioning of aquaporin-positive midbodies thereby influences the direction of cleavage cavity expansion. Notably, disrupting cytokinetic bridge microtubules impairs not only lumenogenesis but also cellular osmoregulation. Our findings reveal a simple mechanism that provides tight spatial and temporal control over the formation of luminal structures and likely plays an important role in water homeostasis during early cleavage stages of a freshwater invertebrate species.

## Introduction

Cytokinetic bridges keep cells interconnected throughout cytokinesis. They contain antiparallel bundles of microtubules that overlap at the midbody, an organelle responsible for recruiting the components required for abscission (Hu et al., 2012; D’Avino et al., 2015; Capalbo et al., 2019). This evolutionary ancient mode of daughter cell separation likely dates back to the last common ancestor of animals and even shares numerous features with those of choanoflagellates, plants and archaeans (Otegui et al., 2005; Eme et al., 2009; Laundon et al., 2019; Yagisawa et al., 2020). In addition to controlling the timing and location of final daughter cell separation, the midbody acts as a polarity cue (Dionne et al., 2015). Many proteins recruited via cytokinetic bridges play dual roles in cytokinesis and apical membrane specification (Román-Fernández and Bryant, 2016). Prior to abscission, positioning of the cytokinetic bridge can thus determine the site of apical domain and apical lumen formation (Frémont and Echard, 2018).

In order to organize cells into tissues, metazoan development relies on lumenogenesis, which can be achieved via diverse mechanisms (Datta et al., 2011). Coupling cytokinesis with the *de novo* generation of intercellular lumens requires the delivery of both, apical determinants and lumen-promoting factors to the cytokinetic bridge. This process appears to be chiefly mediated by endosomes carrying specific Rab GTPases on their surface (Jewett and Prekeris, 2018). Proper trafficking of these Rab endosomes is orchestrated by complex molecular networks that have been extensively investigated in a range of *in vivo* and tissue culture models (Jewett and Prekeris, 2018; Rathbun et al., 2020). However, while numerous targeting regulators have been identified, much less is known about the relevant cargoes transported via different Rab pathways and how they might influence lumen morphogenesis.

Prime candidates for driving luminal expansion, which generally involves redirection of intracellular water to an extracellular space, are aquaporins (AQPs). These channel proteins exist in most living organisms, where they mediate the transport of water and other small solutes across membranes (Campbell et al., 2008; Ishibashi et al., 2017). AQPs thus contribute to diverse physiological processes across cells, tissues and developmental stages (Liu and Wintour, 2005; Day et al., 2014; Martínez and Damiano, 2017) and play a particularly important role in mammalian blastocoel formation (Watson et al., 2004; Offenberg and Thomsen, 2005). Rapid changes to membrane permeability and lumenogenesis generally rely on the agonist-induced and microtubule-mediated redistribution of AQPs from an intracellular vesicular compartment to the general, apical or basolateral plasma membrane (Huebert et al., 2002; Conner et al., 2012; Mazzaferri et al., 2013; Sundaram and Buechner, 2016; Vukićević et al., 2016). It is becoming increasingly clear that not only cytoskeletal activity, but also water flux and hydrodynamics are of fundamental importance for the determination of cell shape, fate, movement and division (Li et al., 2017; Chan et al., 2019; Dumortier et al., 2019). Yet, very little information is available on the sub-cellular localization and functions of AQPs throughout both, early embryogenesis and cytokinetic processes.

Here we explore the dynamic distribution of a maternally inherited AQP during initial cleavage stages of the quagga mussel, *Dreissena rostriformis*, an invasive freshwater bivalve known for its enormous ecological and economic impact (Karatayev et al., 2015; Rudstam and Gandino, 2020). Early dreissenid embryos are especially suited for observing lumenogenesis, since a large intercellular cleavage cavity forms with each blastomere division, to allow for the excretion of excess water in an hypoosmotic environment (Meisenheimer, 1901; Calcino et al., 2019). Furthermore, only a single AQP ortholog, *Dro-lt-AQP1*, is highly expressed in unfertilized eggs and early cleavage stages of *D. rostriformis* (Gene.75921, Figure S18 in Calcino et al., 2019). The lophotrochozoan-specific Dro-lt-AQP1 protein belongs to the classical (i.e., water-selective) AQP subtype (Calcino et al., 2019) and was analyzed with respect to microtubular rearrangements, using immunofluorescence and pharmacological treatments. Our findings reveal a previously undescribed cell biological process that allows precise control over the timing and direction of intercellular lumen formation during cytokinesis by utilizing the ancient molecular machinery that underlies polarized trafficking to the midbody.

## Materials and Methods

### Animals

Adult specimens of the freshwater mussle *Dreissena rostriformis* were collected in the New Danube (Georg-Danzer-Steg, Vienna, Austria, 48°14′44.8″N 16°23′39.3″E) and kept in a large aquarium filled with Danube river water at 18°C. To induce spawning, animals were cleaned with a toothbrush, rinsed with tap water and placed into 2 µm filtered river water. Serotonin (#H9523, Sigma-Aldrich, St. Louis, Missouri, United States) was added at a final concentration of 0.1 mg/ml and the animals were incubated for 20 min at room temperature in the dark. They were then placed into individual glass dishes, where most individuals spawned within 1–2 h of serotonin exposure. Eggs were pooled into a fresh dish, inseminated with a few drops of pooled sperm solution and incubated on a shaker for 30 min. Excess sperm was then washed from fertilized zygotes with several changes of 2 µm filtered river water. Embryos were left to develop at 21°C in the dark and fixed for 1 h in ice-cold 4% PFA (paraformaldehyde, #158127, Sigma-Aldrich) in PBS (0.01 M phosphate buffered saline, #1058.1, Carl Roth, Karlsruhe, Germany) containing 2% acetic anhydride (#CP28.1, Carl Roth GmbH + Co. KG, Karlsruhe, Germany). The samples were then washed three times in PBS and stored at 8°C in PBS containing 0.1% sodium azide (#106688, Merck, Darmstadt, Germany).

### Immunofluorescence

A polyclonal antibody against Dro-lt-AQP1_Gene.75921 was generated by Eurogentec (Seraing, Belgium) using their Speedy Mini immunization program. Specifically, one rabbit was immunized with a synthetic peptide (nh2- C + VIDGKGDFQRLPTEE–conh2) corresponding to amino acids 396–410 of the Dro-lt-AQP1_Gene.75921 protein (Calcino et al., 2019). Following the initial immunization and three subsequent boosters, a pre-immune bleed and a final bleed were obtained. The latter was used for affinity purification. Upon receipt, the purified antibody (in PBS, 0.01% thimerosal and 0.1% BSA) was diluted 1:1 in glycerol (#104201, Merck, Darmstadt, Germany) and 4 µl aliquots were stored at -20°C.

Antibody specificity was assessed by Western Blotting, which revealed a strong band at the expected molecular weight for Dro-lt-AQP1_Gene.75921 protein (∼50 kDA, Supplementary Figure S1E). Pooled eggs of *D. rostriformis* were pelleted, washed twice with 2 µm filtered river water and flash frozen in liquid nitrogen. Samples were stored at -80°C until further processing. Eggs were resuspended in 50 µl RIPA lysis buffer (20 mM 3-(N-morpholino)propanesulfonic acid (MOPS), 150 mM NaCl, 1 mM ethylenediaminetetraacetic acid (EDTA), 1% Nonidet P-40) and 0.1% sodium dodecyl sulfate (SDS)) containing protease and phosphatase inhibitor cocktails (P8340 and P0044, Sigma-Aldrich, Steinheim, Germany). The samples were homogenized with a Tissue Lyser at 45 Hz for 30 s, placed into an ultrasonic bath for 10 s and centrifuged at maximum speed for 15 min. Total protein concentration of the supernatant was quantified using a Bradford protein assay (#5000001, Bio-Rad Protein Assay Kit II, Bio-Rad Laboratories, Hercules, CA, United States). The protein lysate with DTT (100 mM, #1114740001, VWR, Vienna, Austria) and 4x loading buffer (0.3 M Tris base/10% SDS/50% glycerol/0.05% bromphenol blue) was denatured at 95° for 5 min and separated by electrophoresis on a 10% SDS-polyacrylamide gel in electrophoresis buffer (25 mM Tris base/192 mM Glycin/0.1% SDS) at 110 V for 1.5 h. Separated proteins were transferred to an Immun-Blot®-polyvinylidene difluoride membrane (#1620177, Bio-Rad Laboratories, Hercules, CA, United States) using a Trans Blot^®^ Turbo Transfer System (STANDARD SD Program (25V, 1A, 30 min), #1704150, Bio-Rad Laboratories, Hercules, CA, United States). The membrane was dried overnight and nonspecific binding sites were blocked in 5% nonfat dry milk (MMP, A0830.0500, Applichem, Darmstadt, Germany) diluted in tris buffered saline with Tween 20 (TBST; 10 x TBS, 48,4 g Tris base, 160 g NaCl) for 1 h. For immunological detection of Dro-lt-AQP1, the membrane was incubated with the primary antibody diluted 1:200 (in 5% MMP in TBST) at 4°C overnight. Following 3 times washing, incubation with the secondary antibody (#7074 anti-rabbit IgG, HRP-linked Antibody, Cell Signaling, Danvers, MA, United States) diluted 1:5000 in 5% MMP in TBST was carried out at room temperature for 1 h. Following another washing step, protein bands were detected using a luminol-based enhanced chemiluminescence horseradish peroxidase (HRP) substrate (#34075, Super Signal West Dura kit, Thermo Fisher Scientific, Waltham, MA, United States) and the ChemiDoc XRS System (#1708265, Bio-Rad Laboratories, Hercules, CA, United States).

For immunofluorescence, early cleavage stage embryos of *D. rostriformis* were rinsed three times with PBS and incubated for 1 h in blocking solution, i.e., in PBS containing 1% Tween®20 (#9127.1, Carl Roth, Karlsruhe, Germany) and 3% normal goat serum (#PCN5000, Invitrogen, Molecular Probes). The embryos were then incubated overnight at 8°C in the primary antibodies diluted in blocking solution. For this step, our custom Dro-lt-AQP1 antibody (diluted 1:200), anti-acetylated α-tubulin (1:800, mouse, monoclonal, #T6793, Sigma, St. Louis; MO, United States) and anti-tyrosinated α-tubulin (1:800, mouse, monoclonal, #T9028, Sigma, St. Louis; MO, United States) were used. Following six washes with PBS, the embryos were incubated overnight at 8°C in PBS containing the secondary antibodies goat anti-rabbit Alexa Flour 633 (1:500, #A21070, Invitrogen, Molecular Probes) and goat anti-mouse Alexa Flour 488 (1:500, #A11001, Invitrogen, Molecular Probes) as well as the nucleic acid stain Hoechst (1:5000, Sigma-Aldrich; St. Louis; MO, United States). After a final six washes in PBS, specimens were mounted in Fluoromount-G (Southern Biotech, Birmingham, AL, United States) and stored at 4°C. Negative controls were performed by omitting the primary antibodies and yielded no signal (Supplementary Figure S1A–D).

### Nocodazole Treatments

Pharmacological experiments were carried out with pooled embryos from three females and three males and in three technical replicates. Nocodazole (#M1404, Merck KGaA, Darmstadt, Germany) was dissolved in DMSO (#A994.2, Carl Roth GmbH + Co. KG, Karlsruhe, Germany) and stored as 33 mM stock solution at -20°C. Embryos were incubated in 2 µm filtered river water containing 10 µM nocodazole and 42 nM DMSO (= 0.033%). Control embryos were treated with 42 nM DMSO. Treatments were carried out in the dark at 21°C and maintained for the entire duration of the experiments. Embryos were either treated from 45 mpf (minutes post fertilization) or from 1 hpf (hours post fertilization) until fixation at 1.5 hpf and 2 hpf, respectivley.

### Imaging, Volumetric Measurements and Statistical Analyses

Confocal laser scanning microscopy was performed on a Leica TCS SP5 II microscope (DMI6000 CFS, Leica Microsystems, Wetzlar, Germany). Maximum projections of image stacks were generated and global brightness and contrast were adjusted in ImageJ (Schneider et al., 2012).

Volumetric measurements of blastomeres and cleavage cavities were conducted with the software Amira (v. 2020.2, ThermoFisher). Each respective structure was segmented by manual labelling (Figure 4B) and interpolation between sections. Segmented areas were subsequently measured with the Material Statistics tool.

To document nocodazole treatment effects, n > 23 embryos were analyzed for each condition. Raw measurement data is provided in Supplementary Data S1. To compare median cell and cavity volumes between different conditions, a two-sided Wilcoxon rank-sum test was performed with several *p*-value thresholds (****p =< 1e-04, ***p =< 0.001, **p =< 0.01, *p =< 0.05, n. s *p* > 0.05, Figure 4).

## Results and Discussion

### AQP Recruitment to the Cytokinetic Midbody Coincides With Lumen Expansion

**FIGURE 1:**
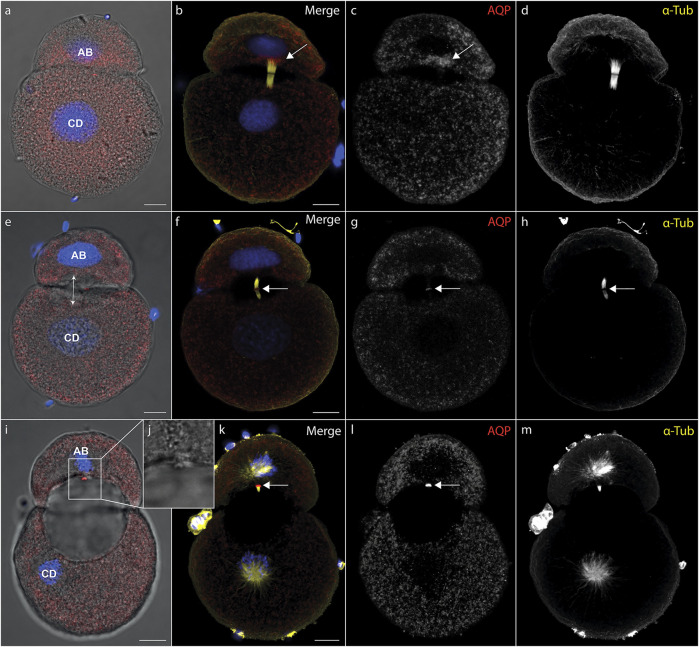
Recruitment of midbody aquaporin and formation of the first cleavage cavity. **(A–l)** Maximum intensity projections of *Dreissena rostriformis* embryos at 1 hpf **(A–D)**, 1.25 hpf **(E–H)** and 1.5 hpf **(I–L)**. Hoechst labeling of nuclei is shown in blue, Dro-lt-AQP1 immunofluorescence is shown in red **(A,B,E,F,I,K)** or grey-scale **(C,G,L)** and acetylated/tyrosinated alpha-tubulin immunofluorescence is shown in yellow **(B,F,K)** or grey-scale **(D,H,M)**. AB and CD blastomere morphology and cleavage cavity expansion are shown in overlays of brightfield images with Dro-lt-AQP1 immunofluorescence **(A,E,I)**. Arrows in **(B,C)** indicate Dro-lt-AQP1 accumulation at the cytokinetic bridge. Arrows in **(F,G,H,K,L)** indicate the location of the Dro-lt-AQP1 immunoreactive midbody. The double-headed arrow in **(E)** indicates the direction of cleavage cavity expansion. **(J)** Brightfield close-up of the Dro-lt-AQP1 immunoreactive midbody remnant shown in **(I)**. Scale bars, 10 µm.

**FIGURE 2:**
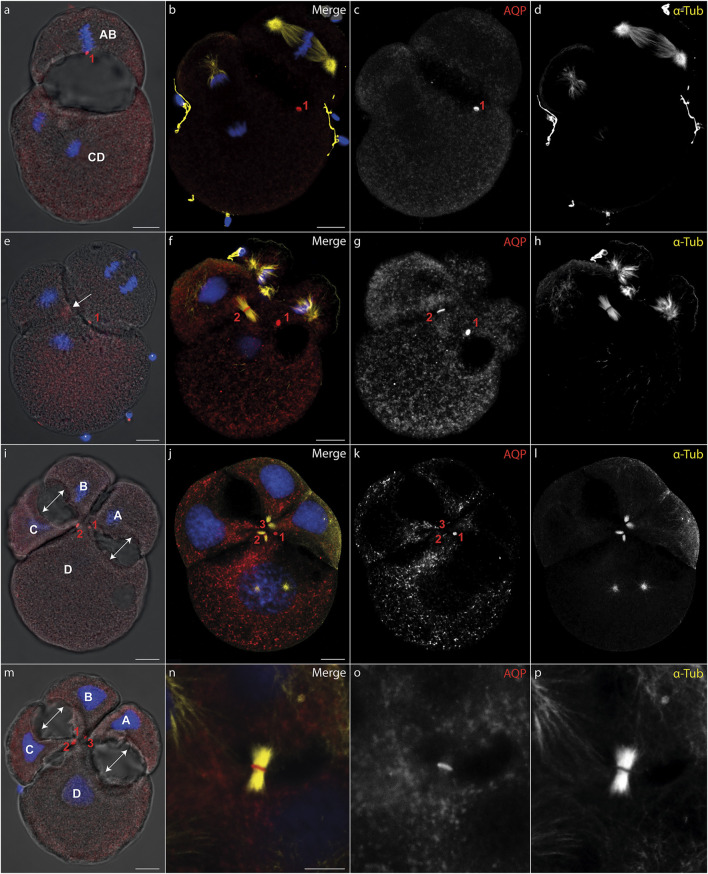
Midbody localization determines the direction of cleavage cavity expansion. **(A–P)** Maximum intensity projections of *Dreissena rostriformis* embryos at 1.5 hpf **(A)**, 1.75 hpf **(B–H)** and 2 hpf **(I-P)**. Hoechst labeling of nuclei is shown in blue, Dro-lt-AQP1 immunofluorescence is shown in red **(A,B,E,F,I,J,M,N)** or grey-scale **(C,G,K,O)** and acetylated/tyrosinated alpha-tubulin immunofluorescence is shown in yellow **(B,F,J,N)** or grey-scale **(D,H,L,P)**. AB and CD blastomere morphology and cleavage cavity expansion are shown in overlays of brightfield images with Dro-lt-AQP1 immunofluorescence **(A,E,I,M)**. Midbodies are numbered in the order of their formation during the first (1) and second cleavages (2 and 3). The arrow in (**e**) indicates Dro-lt-AQP1 accumulation prior to formation of the second midbody between blastomeres C and D. Double-headed arrows in **(I,M)** indicate the direction of cleavage cavity expansion. **(N–O)** Close-up of a cytokinetic bridge and Dro-lt-AQP1 immunoreactive midbody. Scale bars, 10 µm.

During cytokinesis, spindle microtubules become partially reorganized into a cytokinetic bridge, which can be observed particularly well in early cleavage stages of *D. rostriformis* (Figures 1, 2). Maternally inherited Dro-lt-AQP1 is then recruited to this cytokinetic bridge (Figures 1B–D, Figure 2E, arrows). Dro-lt-AQP1 accumulation at the midbody coincides with the onset of cleavage cavity formation (Figures 1A–H, Figures 2F–I). While the cleavage cavity expands, Dro-lt-AQP1 immunoreactivity increases within the midbody (Figures 1G,L). Importantly, however, Dro-lt-AQP1 is otherwise absent from the plasma membrane. Once abscission is completed (Figures 1I–L), the Dro-lt-AQP1-immunoreactive midbody is inherited by one of the two daughter cells and persists within its membrane, at least until the four-cell stage (Figures 2A–M). The tubulin fibers of the cytokinetic bridge, in contrast, dissolve rapidly and the cleavage cavity collapses (Figures 2B–E), expelling its contents to the exterior. Although numerous studies have addressed AQP recruitment to specific plasma membrane domains (Mazzaferri et al., 2013; Vukićević et al., 2016; Arnspang et al., 2019) as well as their emerging roles in cell proliferation and cancer biology (Galán-Cobo et al., 2016; Dajani et al., 2018), detailed analyses of the sub-cellular localization of AQP during early embryonic development and during cytokinesis are currently lacking. Accordingly, this is the first report, to our knowledge, of AQP recruitment to the midbody.

The two-cell stage of *D. rostriformis* consists of a smaller AB blastomere and a larger CD blastomere (Figure 1) (Meisenheimer, 1901). Interestingly, the larger CD blastomere divides slightly earlier than the smaller AB blastomere, giving rise to a transient three-cell stage (Figures 2B–H). The second round of cleavages results in two additional cytokinetic bridges that do not form centrally between the dividing A/B and C/D blastomeres, but instead are displaced towards the interface between the A/D and B/C cousin blastomeres in the center of the embryo (Figures 2E–M). Consequently, the two new cleavage cavities form between these cousin blastomeres and not between the A/B and C/D daughter blastomeres (Figures 2I,M, double-headed arrows). This is consistent with vertebrate studies linking cytokinetic bridge and midbody positioning to the site of lumen formation (Rodriguez-Fraticelli et al., 2010; Klinkert et al., 2016; Frémont and Echard, 2018; Rathbun et al., 2020).

Since the CD blastomere divides first, Dro-lt-AQP1 accumulation in the C/D cytokinetic midbody precedes that in the A/B cytokinetic midbody (Figures 2E–I). However, the remnant of the Dro-lt-AQP1-immunoreactive midbody from the first cleavage (Figure 2, red “1”) might compensate for this time lag, since the two new cleavage cavities expand almost simultaneously (Figures 2I,M). Notably, the midbody remnant gradually shifts towards the newly formed Dro-lt-AQP1-immunoreactive midbodies in the center of the developing embryo (Figures 2I–M). This likely allows for a precise temporal and spatial control over the water efflux from each blastomere, since we observed no Dro-lt-AQP1 accumulation in other areas of the embryos’ cell membranes. Midbody remnants have been shown to influence multiple postmitotic processes, including lumenogenesis, cell proliferation, cell signalling, cell polarity and fate specification as well as the formation of polarized structures such as neurites and cilia (Antanavičiūtė et al., 2018; Peterman and Prekeris, 2019; Labat-de-Hoz et al., 2021). As such, there is a noteworthy overlap with known roles of AQPs not only in lumenogenesis (Huebert et al., 2002; Hashizume and Hieda, 2006; Ferrari et al., 2008; Khan et al., 2013), but also in the regulation of cell stemness and proliferation (Galán-Cobo et al., 2016; Dajani et al., 2018; Jung et al., 2021).

Our findings show that intercellular lumenogenesis in early cleavage stages of *D. rostriformis* is likely mediated by Dro-lt-AQP1 localized in the midbody and midbody remnant. Given their above-mentioned multifunctional properties, this close association between midbodies and AQPs may have important implications for various cellular events, warranting further investigations.

### Depolymerisation of Cytokinetic Bridge Microtubules Triggers Ectopic AQP Translocation and Impairs Both Lumenogenesis and Osmoregulation

Our next aim was to prevent Dro-lt-AQP1 targeting to the midbody in order to assess its potential involvement in lumenogenesis and osmoregulation. For early animal cleavage stages, 10 µM nocodazole has been shown to be sufficient to completely depolymerize spindle microtubules (Chenevert et al., 2020). Such treatments can have confounding effects, since they interrupt cellular trafficking. However, nocodazole is widely used in cell biology studies and is not known to impair cellular osmoregulation [i.e., no significant increase in cell volume and no significant effect on a cells ability to recover from hypoosmotic shock, e.g., see (Fernández and Pullarkat, 2010)]. Furthermore, we timed our experiments to specifically target the period of midbody formation and to minimize the duration of drug exposure.

**FIGURE 3:**
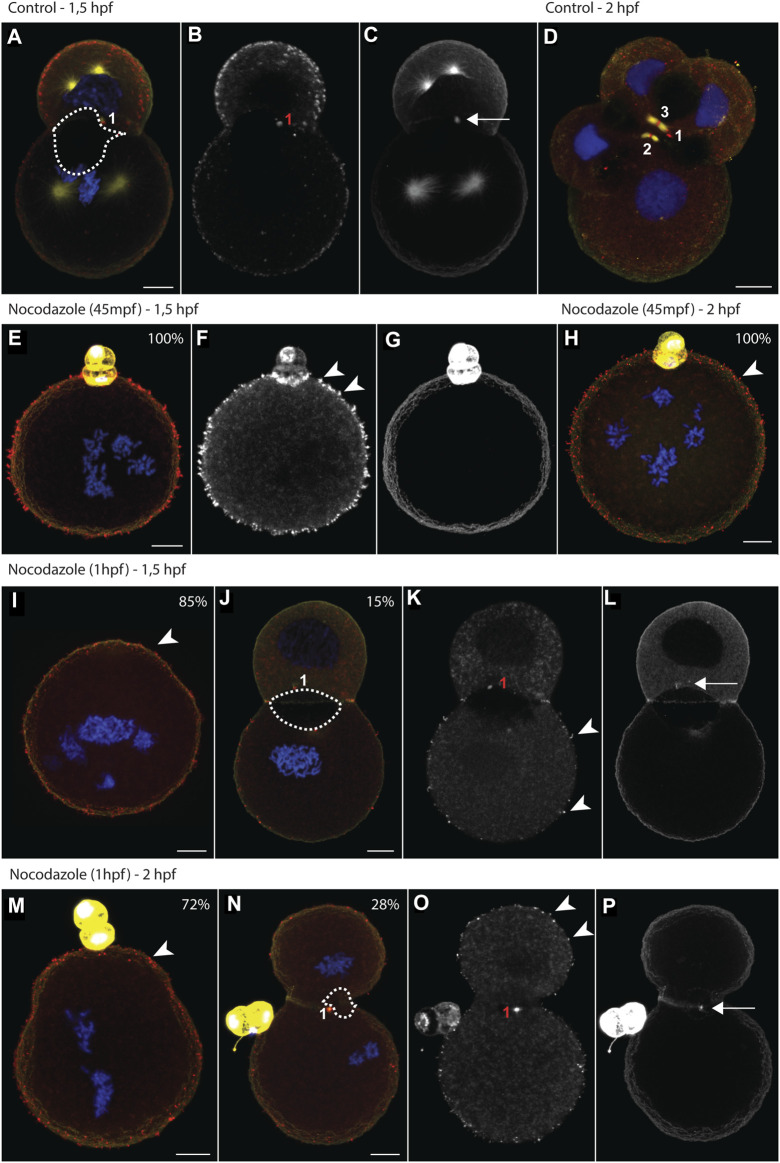
Nocodazole treatments trigger aquaporin translocation and impair lumenogenesis. **(A–P)** Maximum intensity projections of *Dreissena rostriformis* embryos at 1.5 hpf **(A–C, E–G, I–L)** and 2 hpf **(D,H,M–P)**. Embryos were treated with 10 µM nocodazole either from 45 mpf **(E–H)** or from 1 hpf onwards **(I–P)**. Values in the upper right corner of an image indicate the percentage of embryos with the depicted phenotype for the respective treatment condition. Hoechst labeling of nuclei is shown in blue, Dro-lt-AQP1 immunofluorescence is shown in red **(A,D,E,H,I,J,M,N)** or grey-scale **(B,F,K,O)** and acetylated/tyrosinated alpha-tubulin immunofluorescence is shown in yellow **(A,D,E,H,I,J,M,N)** or grey-scale **(C,G,L,P)**. Midbodies are numbered in the order of their formation during the first (1) and second cleavages (2 and 3). The dotted outline in **(A,J,N)** indicates the maximal expansion of the cleavage cavity. Arrows indicate remnants of the cytokinetic bridge **(C,L,P)** and arrowheads point to Dro-lt-AQP1-immunoreactive membrane protrusions **(F,H,I,K,M,O)**. Scale bars, 10 µm.

One-cell stage embryos of *D. rostriformis* were exposed to nocodazole either from 45 mpf (minutes post fertilization), i.e., after nuclear division but prior to cytokinetic bridge formation, or from 1 hpf (hours post fertilization), i.e., from early stages of cytokinetic bridge and midbody formation. Drug treatments were maintained for 30, 45, 60 or 75 min. Afterwards, the embryos were fixed either at the two-cell stage (1.5 hpf) or at the four-cell stage (2 hpf) (Figure 3). For each condition, the exact number of specimens analyzed (n > 20) and all raw data are provided in Supplementary Data S1.

**FIGURE 4:**
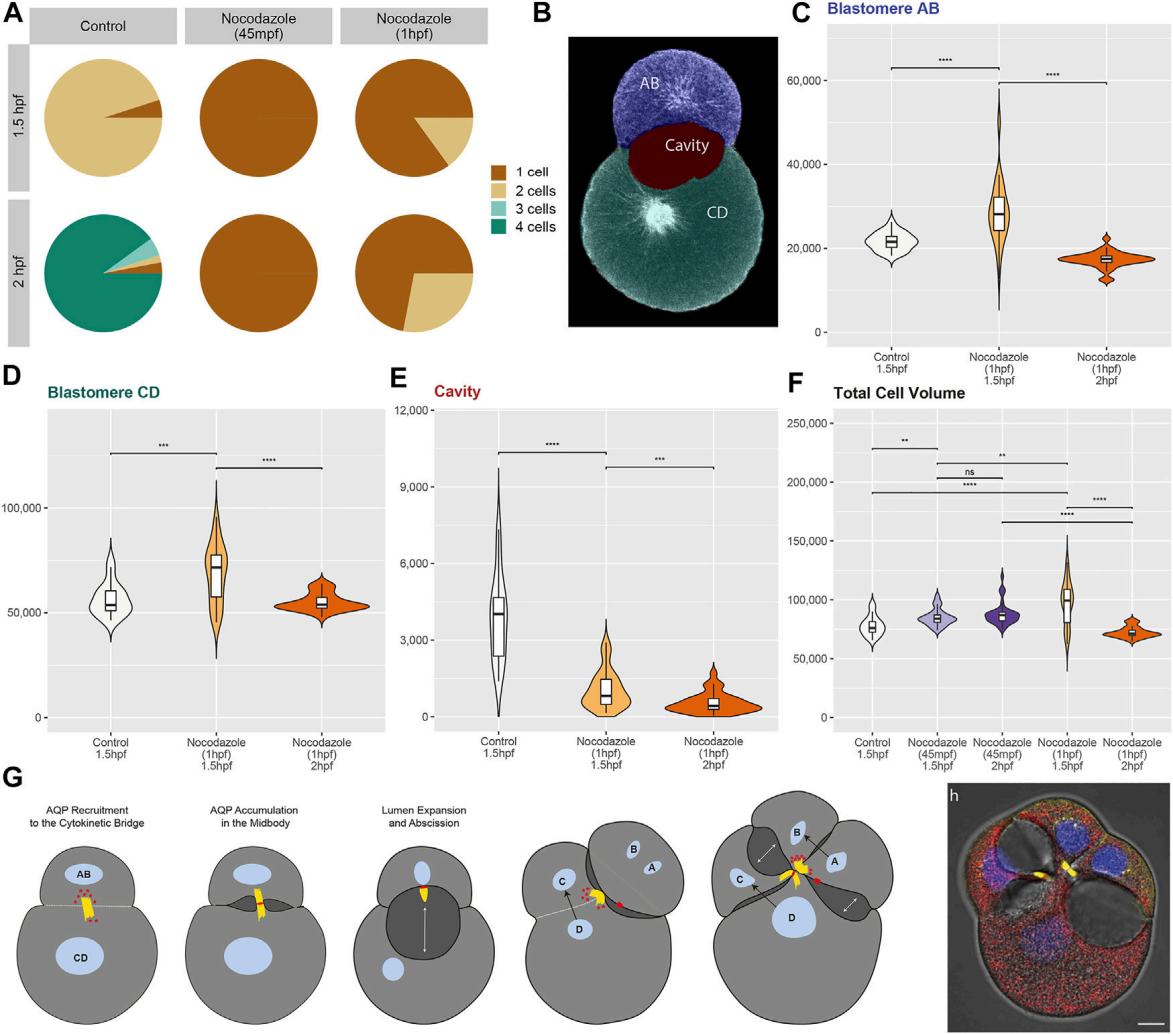
Statistical analyses of nocodazole phenotypes and model of aquaporin function in *Dreissena rostriformis*. **(A)** Pie charts depict the percentage of one-, two-, three- and four-cell stages for embryos at 1.5 and 2 hpf that were exposed to different treatment conditions as indicated. **(B)** Single optical section with areas used for image segmentation and volumetric measurements colorized. **(C–F)** Violin plots illustrate volume distributions in µm³ for the AB blastomere **(C)**, the CD blastomere **(D)**, the cleavage cavity **(E)** and the total cell volume (i.e., AB + CD volumes) **(F)** of embryos exposed to different treatment conditions. For nocodazole treatments from 1hpf, only embryos with a two-cell stage phenotype were analyzed. To compare different conditions, a two-sided Wilcoxon rank-sum test was used with several *p*-value thresholds (****p =< 1e-04, ***p =< 0.001, **p =< 0.01, *p =< 0.05, n. s *p* > 0.05). Comparisons were only made between embryos that are either at the same stage, have the same number of blastomeres or were exposed to the same treatment. **(G)** Schematic representation of the here-proposed model of lumenogenesis. Recruitment of AQP (red) via a cytokinetic bridge (yellow) to the midbody leads to the formation of cleavage cavities (dark grey). Black arrows indicate the direction of blastomere division and double-headed white arrows indicate the direction of cleavage cavity expansion. **(H)** Overlay of a brightfield image and a maximum intensity projection of a control embryo at 2 hpf labeled for Dro-lt-AQP1 immunoreactivity (red), acetylated/tyrosinated alpha-tubulin immunoreactivity (yellow) and Hoechst nuclear staining (blue). Scale bar, 10 µm.

Nocodazole treatment at 45 mpf inhibits cytokinesis entirely (Figures 3E–H, Figure 4A). By 1.5 hpf, 95% of the control embryos are at the two-cell stage (Figures 3A–C), whereas 100% of the treated embryos remain at the one-cell stage (Figures 3E–G, Figure 4A). By 2 hpf, 90% of the control embryos are at the four-cell stage (Figure 3D), whereas 100% of the treated embryos still remain at the one-cell stage (Figure 3H, Figure 4A). One-cell stage embryos lack a cytokinetic bridge and a Dro-lt-AQP1-immunoreactive midbody that could mediate water excretion. However, nocodazole treatment at 45 mpf triggers the ectopic formation of Dro-lt-AQP1-immunoreactive membrane protrusions that are not present in control embryos (Figures 3E–H, arrowheads). Such protrusions can be caused by AQP-mediated water effluxes from a cell (Karlsson et al., 2013), which is consistent with our observation that the total cell volume of embryos treated at 45 mpf increases only slightly (but significantly) compared to control embryos (Figure 4F). Accordingly, ectopic translocation of AQP to the cell membrane likely allows for a limited compensation of osmotic water influx, preventing cell swelling beyond a certain point and osmotic lysis.

This is consistent with data from vertebrates, where various triggers have been shown to induce the reversible sub-cellular translocation of AQPs in order to maintain water homeostasis (Conner et al., 2013). However, it remains to be determined whether the observed Dro-lt-AQP1 redistribution is a natural response to increased hypoosmotic stress or due to nocodazole-induced disruption of polarized membrane trafficking. It should further be noted that early treated embryos are unable to revert to their original volume at later stages (2 hpf, Figure 4F), which shows that their osmoregulation capacity is long-term impaired.

Nocodazole treatment from 1 hpf results in two phenotypes (Figures 3I–P, Figure 4A). At 1.5 hpf, 15% of the embryos are at the two-cell stage (Figures 3J–L), while the rest remain at the one-cell stage (Figure 3I). By 2 hpf, the number of embryos at the two-cell stage increases to 28%, while the rest still remain at the one-cell stage (Figures 3M–P). The two-cell stage embryos show a Dro-lt-AQP1-immunoreactive midbody remnant and a small cleavage cavity (Figures 3J–L, N–P). At 1.5 hpf, the cell volume of both their AB and CD blastomeres is significantly increased (Figure 4C). Furthermore, embryos at 1.5 hpf show a significantly higher total cell volume if treated from 1 hpf than if treated from 45 mpf onwards (Figure 4F). This is likely because they had 15 min less time to react to nocodazole exposure, e.g., by forming Dro-lt-AQP1-immunoreactive membrane protrusions (Figures 3F,I–K arrowheads). The cleavage cavity volume of late treated embryos at both 1.5 and 2 hpf is significantly decreased (Figure 4E), indicating reduced water excretion into this intercellular space after cytokinetic bridge disruption. Importantly, however, embryos at 2 hpf show a significantly lower total cell volume, if treated from 1 hpf compared to if treated from 45 mpf (Figures 3H,N–P, Figure 4F). Reversal of the initial cell volume increase in late treated embryos shows that their osmoregulation capacity is not long-term impaired (as in early treated embryos) but only briefly interrupted, when cytokinetic bridge and midbody formation is not prevented but only partially disrupted (Figure 4F).

These data illustrate how cytokinetic bridge disruption impairs lumenogenesis and cellular osmoregulation, although the latter was partially restored through subsequent translocation of Dro-lt-AQP1 to the cell membrane. We further show that presence of at least an incomplete Dro-lt-AQP1-immunoreactive midbody remnant after cytokinetic bridge depolymerization greatly improves the ability of embryos to compensate for hypoosmotic water influx. Accordingly, since Dro-lt-AQP1 is the only water channel expressed in early developmental stages of *Dreissena rostriformis* (Gene.75921, Figure S18 in Calcino et al., 2019) and exclusively detected in the cytosol (storage) and in the midbody of untreated embryos (Figures 1, 2), we argue that midbody-localized Dro-lt-AQP1 plays a central role in cleavage cavity formation.

In sum, we propose a novel mode of lumenogenesis (Figures 4G,H) that involves AQP recruitment specifically to the midbody during cytokinesis. Temporal and spatial control over cellular water release is likely achieved through placement, inheritance and maintenance of AQP-containing midbodies and midbody remnants. The large cleavage cavities of the quagga mussel, *Dreissena rostriformis,* are an adaptation to freshwater habitats (Calcino et al., 2019). However, the relatively simple mechanism underlying their controlled formation is likely to be widespread among Metazoa. As summarized in Supplementary Data S2, AQPs are present in the zygotes and initial cleavage stages of all investigated species, ranging from cnidarians to vertebrates. While AQPs may serve various functions in these different embryos, they have been implicated in blastocyst cavity formation in mouse (Barcroft et al., 2003; Offenberg and Thomsen, 2005; Frank et al., 2019). Moreover, previously published AQP immunostainings in mammalian oocytes and embryos from zygote to bastocyst stages actually appear to show labelling of cytokinetic bridge- and midbody-like structures that have not been addressed (Xiong et al., 2013; Park et al., 2014; Park and Cheon, 2015). We therefore suggest that the here-described mechanism of lumenogenesis, via AQP-recruitment to the cytokinetic midbody, may be critical for early animal embryogenesis and should be investigated in more taxa.

## Data Availability

The original contributions presented in the study are included in the article/Supplementary Material, further inquiries can be directed to the corresponding authors.
