# Supplementary figures and images for: Midbody-Localized Aquaporin Mediates Intercellular Lumen Expansion During Early Cleavage of an Invasive Freshwater Bivalve
Source: Front Cell Dev Biol. 2022 Jun 14;10:894434. doi: 10.3389/fcell.2022.894434 (PMC9237387; doi:10.3389/fcell.2022.894434)

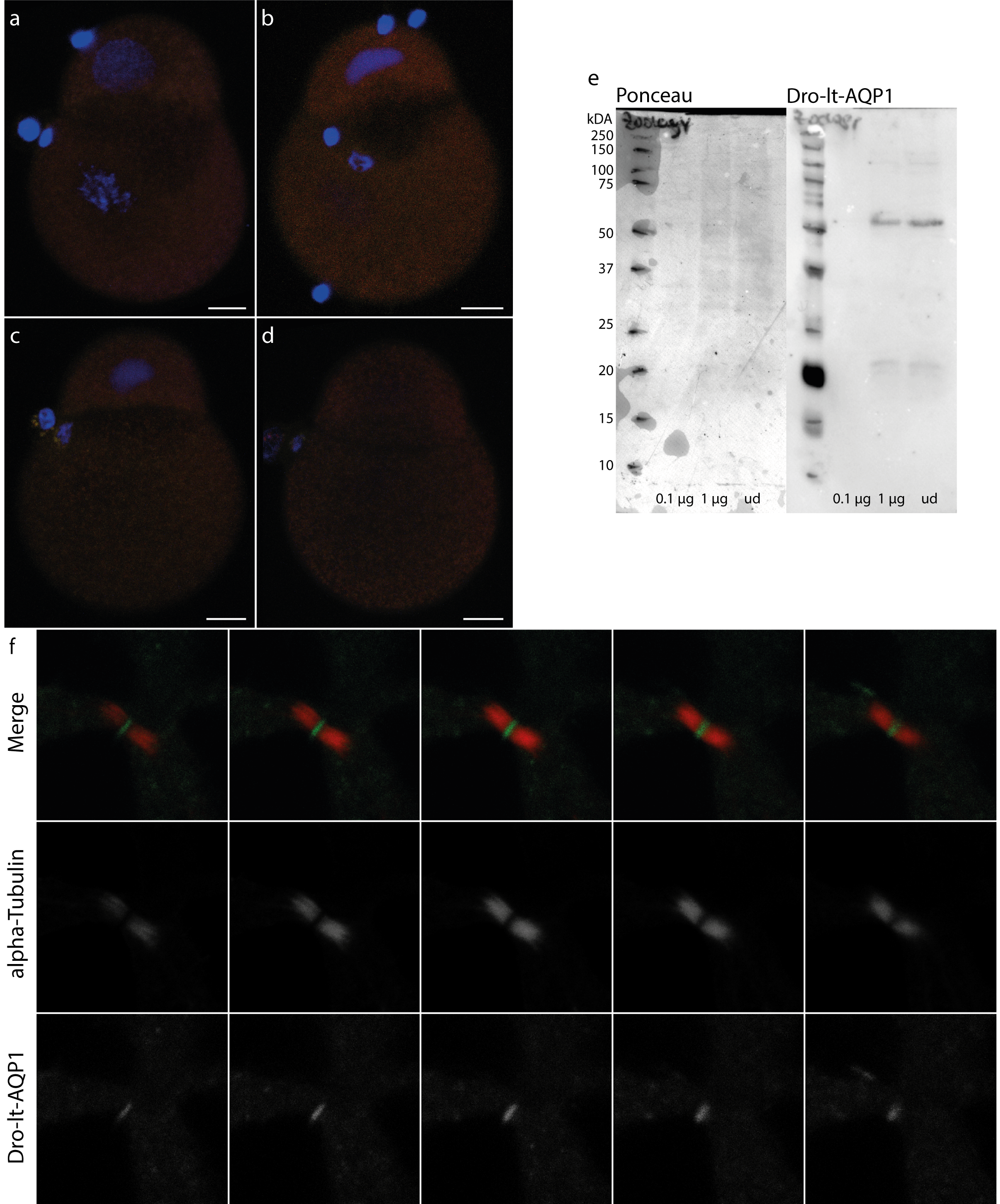

Supplement: Supplementary file 2 [file Image1.TIF]
